# Analysis of a Vaping-Associated Lung Injury Outbreak through Participatory Surveillance and Archival Internet Data

**DOI:** 10.3390/ijerph18158203

**Published:** 2021-08-03

**Authors:** Yulin Hswen, Elad Yom-Tov

**Affiliations:** 1 Department of Epidemiology and Biostatistics, University of California at San Francisco, San Francisco, CA 94158, USA; yuh958@mail.harvard.edu; 2 Bakar Computational Health Sciences Institute, University of California at San Francisco, San Francisco, CA 94143, USA; 3 Innovation Program, Boston Children’s Hospital, Harvard Medical School, Boston, MA 02115, USA; 4 Microsoft Research Israel, 3 Alan Turing Str., Herzeliya 4672415, Israel; 5 Faculty of Industrial Engineering and Management, Technion, Haifa 3200000, Israel

**Keywords:** vaping, electronic cigarette, participatory surveillance, internet data

## Abstract

The US Centers for Disease Control and Prevention alerted of a suspected outbreak of lung illness associated with using E-cigarette products in September 2019. At the time that the CDC published its alert little was known about the causes of the outbreak or who was at risk for it. Here we provide insights into the outbreak through analysis of passive reporting and participatory surveillance. We collected data about vaping habits and associated adverse reactions from four data sources pertaining to people in the USA: A participatory surveillance platform (YouVape), Reddit, Google Trends, and Bing. Data were analyzed to identify vaping behaviors and reported adverse events. These were correlated among sources and with prior reports. Data was obtained from 720 YouVape users, 4331 Reddit users, and over 1 million Bing users. Large geographic variation was observed across vaping products. Significant correlation was found among the data sources in reported adverse reactions. Models of participatory surveillance data found specific product and adverse reaction associations. Specifically, cannabidiol was found to be associated with fever, while tetrahydrocannabinol was found to be correlated with diarrhea. Our results demonstrate that utilization of different, complementary, online data sources provide a holistic view of vaping associated lung injury while augmenting traditional data sources.

## 1. Introduction

On 6 September 2019, the US Centers for Disease Control and Prevention (CDC) put out an Investigation Notice concerning a suspected outbreak of lung illness associated with using e-cigarette products [1]. According to the notice, at the time, there were reports from 33 states of lung illnesses in people who reported to using e-cigarettes or vaping products.

E-cigarettes are products which allow users to inhale aerosolized substances such as nicotine, tetrahydrocannabinol (THC), and cannabidiol (CBD) [2]. They are, since 2014, the most commonly used tobacco product among youths, with 27.5% of high school students and 10.5% middle school students reporting to use them as of 2019 [3].

At the time that the CDC published its Investigation Notice, little was known about the causes of the outbreak or who was at risk for it. Specifically, because of the limited number of cases, the products and ingredients causing the harm, risky usage patterns, and the demographic most likely to be affected were unknown. In this outbreak, as well as similar public health emergencies, finding this information using traditional data sources (as was eventually done in the case of vaping) requires significant effort in investigation time and cost. Moreover, realizing that an outbreak is unfolding is not a trivial undertaking. E-cigarettes are regulated in the US as a tobacco product, specifically in the areas of advertising, child-safety, health warning labelling, minimum age, and reporting [4]. Nevertheless, when seemingly disconnected cases appear in different locations across the country, understanding the link between them and realizing their commonality is challenging [5].

The internet is now used by the majority of the US population [6]. Interactions with the digital environment reflect real-world behaviors in everyday lives and provide a snapshot of our health, allowing study of the latter through the data people create while browsing the internet [7,8,9]. Indeed, disease outbreaks with seemingly disconnected cases stemming from co-location of people during mass gatherings were tracked using internet data [5]. More generally, digital surveillance thorough analysis of user information has been effective at early detection and prevalence estimation of epidemic outbreaks [10]. The most extensively researched illness in this area is seasonal influenza. Unfortunately, one of the most well-known efforts in this area, Google Flu Trends, mispredicted influenza-like illness rates in the US during the 2012–2013 season [11]. However, researchers have significantly improved these models and their predictive performance [12].

Internet search engines such as Bing have also been used to facilitate the early identification of drug abnormalities that lead to future drug recalls [13]. Other platforms that contain more individual-level information such as anonymous online forums (e.g., Reddit), have been shown useful for understanding illicit behaviors such as marijuana and opioid use [14]. Finally, participatory surveillance platforms-approaches that leverage online survey technology with syndromic surveillance through volunteer reporting are used across multiple countries [15,16,17,18] to track emerging disease-related trends [19].

Vaping has been studied through the lens of Internet data. Social media data was the source of several studies, including Twitter [20,21], JuiceDB (a social media platform for people who vape) [22], YouTube [23], Reddit [24], and mobile apps [25]. The health effects of vaping were examined in Reddit data by Chen et al. [26] and in JuiceDB by Li et al. [22].

We note that, in retrospect, the current outbreak received significant awareness and several studies focused on characterizing the outbreak [27], mechanism [28,29,30], and public health response [31]. However, our study illustrates the use of internet data to provide insights into the outbreak in near real-time using diverse data sources.

Thus, few studies examined the adverse effects of vaping or analyzed internet data from more than one source. The unique contribution of our work is that we examine multiple data sources (both Internet and participatory surveillance) for the health effects of vaping during a public health outbreak related to the use of such products.

Each of the above-mentioned Internet sources has the potential to provide a unique vantage point of emerging trends, together contributing to a holistic understanding of a public health emergency. Unfortunately, practical limitations mean that most investigations of health-related issues through internet data are constrained to utilizing a single data source. Therefore, the purpose of this study is to undercover possible routes to E-cigarette or Vaping Associated Lung Injury (EVALI) through four different internet data sources, each contributing a unique angle to the study, and together providing a deep understanding of the illness. More broadly, our goal is to describe a methodology for investigating a public health outbreak through passive and participatory data sources.

## 2. Materials and Methods

### 2.1. Data Sources

We extracted data from four separate internet data sources. These sources differ in their reach, coverage, granularity, volume, and method of generation. A summary of the data sources appears in Table 1.

The first source, YouVape, was deployed only after the outbreak was known and collected specific information pertaining to the outbreak. The second data source, Google Trends, provides high level measures of query popularity across time and location. We used this data source to inform of the geographic spread of products and of their relative popularity in the population. Data from this source was accessed from more than a year prior to the outbreak and serves also as a baseline for the popularity of these products before their potentially harmful effects were known. Our third data source is Bing, where queries at the individual level were analyzed. These data are known to be useful for identifying adverse reactions in medical drugs and in vaccines [32] and could thus potentially be used to identify harms caused by vaping. However, to minimize the potential effect of news reports, we utilized these data for the nine months prior to the outbreak. Finally, a social media source, Reddit, was used to complement Bing data, as these data are more detailed (long posts, compared to short query texts).

In all data sources we focused on the most popular legal and illicit vaping products and brands on the current market on the market. These popular vaping brands were identified exploratory investigation of forums, social media and blogs and further supplemented by users’ responses on YouVape about the vaping brands they most frequently used. The brand identified included the following: blu, brass knuckles, cereal carts, dank vape, exotics, juul, kingpen, mario carts, mig21, pax, stiiizy, and TKO.

#### 2.1.1. Source 1: YouVape

YouVape is a real-time participatory surveillance platform (https://www.YouVape.org) that seeks to identify health symptoms associated with vaping-related behaviors and was developed by Boston Children’s Hospital, Harvard Medical School. Health symptoms of e-cigarette or vaping product use associated with lung injury (EVALI) was based on clinical symptoms of EVALI. On this self-reporting Internet platform, volunteering users answered sociodemographic, geographic, vaping-related behavioral questions and medical symptoms. Therefore, a non-probability based voluntary sampling method was used which consisted of users who self-selected themselves into the participatory surveillance system, YouVape.

Recruiting to the platform was achieved by creating a press release describing the platform and encouraging people to share their experiences on it. This release was cited widely by news outlets and several healthcare websites.

Thus, users are self-selected and are likely more strongly interested and invested in understanding vaping and its links with EVALI compared to the general population.

#### 2.1.2. Source 2: Google Trends

Google trends (https://trends.google.com, accessed 16 February 2020) is a publicly available platform by Google that provides cumulative information on the volume of queries for selected search terms [33]. Google trends provides a relative search volume for selected queries by analyzing the fraction of total Google web searches over a period of time to estimate the search volume for the selected queries. Here, relative means that the query volumes are scaled between 0 and 100, depending on the specific query issued to Google Trends. For this study we restricted the timeline from 1 January 2018 to 1 January 2020.

#### 2.1.3. Source 3: Reddit

Reddit (https://www.reddit.com, accessed 15 February 2020) is a popular social network organized around communities of shared interests known as ‘subreddits’. We extracted all postings made until 31 December 2019 to the “Vaping101” subreddit, defined as a subreddit “for people to get information when they’re just starting out on their vaping career”. We then extracted all postings to any subreddit made by users who posted to Vaping101.

We hypothesize that the first post by users on this subreddit is an indication of the date that they began their use of vaping products, given the declared goal of this group. We focus on first-time users so as to distinguish between ongoing health experiences and ones which may be due to the onset of vaping.

Thus, for each of the latter postings we computed their posting date relative to the first posting by the user in the Vaping101 subreddit.

Symptom mentions were identified by searching the text of the postings for 161 symptom keywords and their (validated) colloquial synonyms, according to the list compiled by Yom-Tov and Gabrilovich [32]. Synonyms were grouped to their symptom keyword. Symptoms were scored as the relative frequency of their mention by users after their first post on Vaping101, compared to before it.

Age and gender information were identified by finding those posts which contained this information in the popular Reddit representation for these data, e.g., “[M19]” for 19-year-old male. Only posts where a single identifier was matched were retained so as to remove instances where multiple people were mentioned in the post.

#### 2.1.4. Source 4: Bing

We extracted nine months (October 2018–June 2019) of searches made to the Bing search engine by people in the United States. Each record comprises of the text of the search, its time and date, the US state from which it was made, and an anonymous user identifier.

Queries were matched for symptoms as described above for Reddit data. Queries related to vaping were identified by finding in the query text mentions of the vaping products listed above, as well as the following generic keywords: e-cig, electronic cigarette, e-cigarette, vaporizer, vaping. Users who mentioned one of these keywords were included in the vaping group. To facilitate the analysis of adverse reactions in the group which mentioned vaping products we followed the methodology of Yom-Tov and Gabrilovich [32] and defined the control group as a random sample of users who did not search for vaping products but searched for one of the symptoms (see Table 1 for group sizes).

For queries by users in the population who mentioned one of these vaping products we used the posting date of each query relative to the first time that they mentioned a vaping product. Relative time for users who only mentioned symptoms were computed relative to a random date, following Yom-Tov and Gabrilovich [32], and the symptoms were then scored using the QLRS chi-like procedure developed therein. Specifically, a 2 × 2 matrix was computed, where the rows of the matrix are the number of people in the vaping group or the controls and the columns the number of people who queried for the symptom at relative time less than zero or greater than zero. QLRS is the chi-square score of this matrix.

### 2.2. Analysis Overview

Summary statistics of usage are provided for all sources. The association between product use and demographics and geography are analyzed using chi^2^ tests.

The likelihood of reporting each of the adverse reactions on YouVape and Bing was analyzed using a logistic regression model.

## 3. Results

### 3.1. Population Statistics

**Figure 1:**
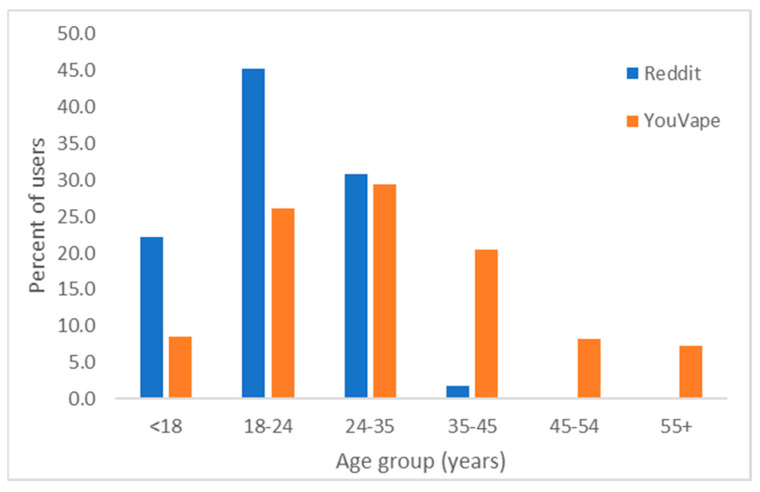
Age group distribution on Reddit and on YouVape.

Table 1 shows the number of users identified in each of the four data sources used. Two data sources (Reddit and YouVape) contained age and gender data for some of the users (Reddit *n* = 117, YouVape *n* = 716). On average, 78% of Reddit users and 74% of YouVape were males. Their age distribution is shown in Figure 1. The distributions are statistically significantly different (*p* < 0.001, chi^2^ test), with Reddit users being younger than YouVape users.

Approximately 62% of YouVape users reported beginning vaping more than one year prior to their report, 14% within 6–12 month, 19% within the last 6 months, and the remainder in the past month. Asked when they last vaped, 62% reported in the past week, 17% within the past month, and the remaining more than 1 month prior; 57% reported vaping more than 3 times per day, 10% vaped 2–3 times per day, 9% once per day, 19% less than once per day, and the remaining did not report their vaping frequency. Adverse reactions were reported by 60% of people. Duration of vaping was associated with duration of reported adverse reactions (chi^2^ test, chi = 66.0883, *p* < 0.00001).

Vaping products were bought from convenience stores or gas stations (28%), family or friends (23%), online (30%), or pharmacies (7%). Additionally, 70 users (9.7%) reported that made their own homemade vaping liquid (e-juice). Out of the 437 (60.7%) of users that selected that they use “other” brands listed, 59 (13.5%) of these users also stated that they made their own homemade vaping liquid. Users from YouVape had the opportunity to describe the ingredients they used of their homemade vaping liquid. Out of the 44 YouVape users that listed their homemade vaping liquid ingredients, 36 (82%) used vegetable glycerin, propylene glycol, nicotine base (Nbase), or flavorings. Other ingredients users mentioned include aroma, pure grain alcohol, marijuana extract, DMT (N, N-Dimethyltryptamine), and 3-MEO-PCP (3-Methoxyphencyclidine).

On Reddit, 34 users mentioned vegetable glycerin, 33 propylene glycol, 27 mentioned nicotine base (Nbase), 2 mentioned pure grain alcohol and 2 marijuana extract, 61 referred to DMT (N, N-Dimethyltryptamine), and 4 to 3-MEO-PCP (3-Methoxyphencyclidine). We note that a specific subreddit (/r/DIY_eJuice) is devoted to homemade vaping recipes, but it was not analyzed in this work.

**Figure 2:**
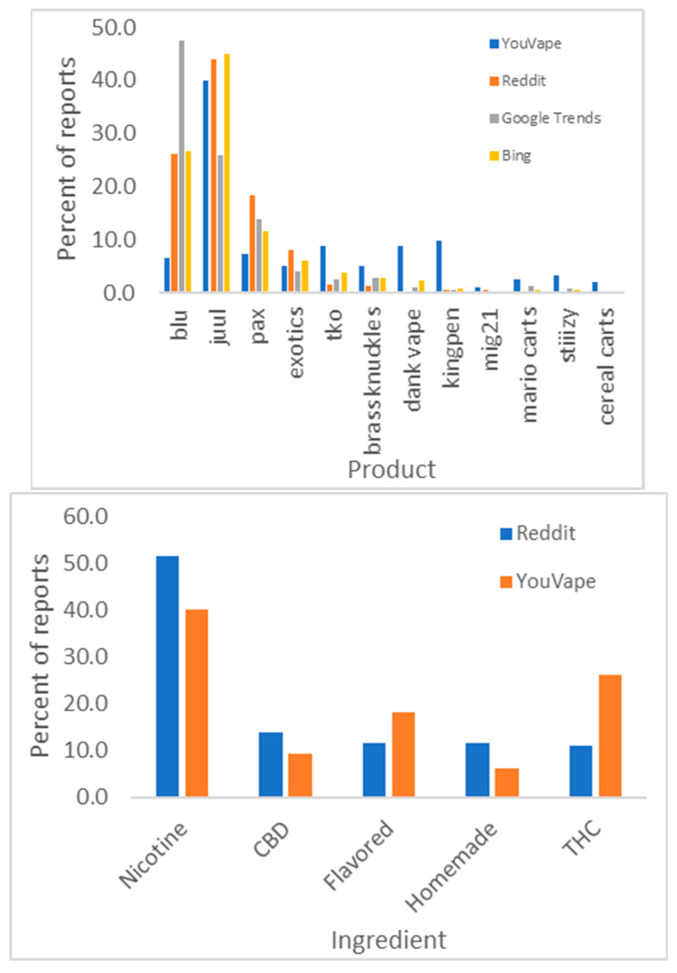
Product popularity in different data sources (**top**) and vaping ingredients (**bottom**).

The most commonly reported products on Reddit were (in descending order) Juul, blu, and pax, and the most common ingredients nicotine and THC (see Figure 2). Pairwise correlations between product popularities are statistically significant (*p* < 0.05 with Bonferroni correction) for Reddit, Google Trends, and Bing. The correlation with YouVape is not statistically significant. The biggest disparity in product popularity is “blu”. There is a good correspondence between Reddit and YouVape in ingredients reported.

YouVape data shows that brand use is strongly associated with age (chi^2^ test, chi = 140.6, *p* = 10^−7^) but not gender (*p* = 0.3). Conversely, ingredients are not correlated with age (*p* = 0.11), but are associated with gender (chi^2^ test, chi = 56.8, *p* = 10^−10^).

**Figure 3:**
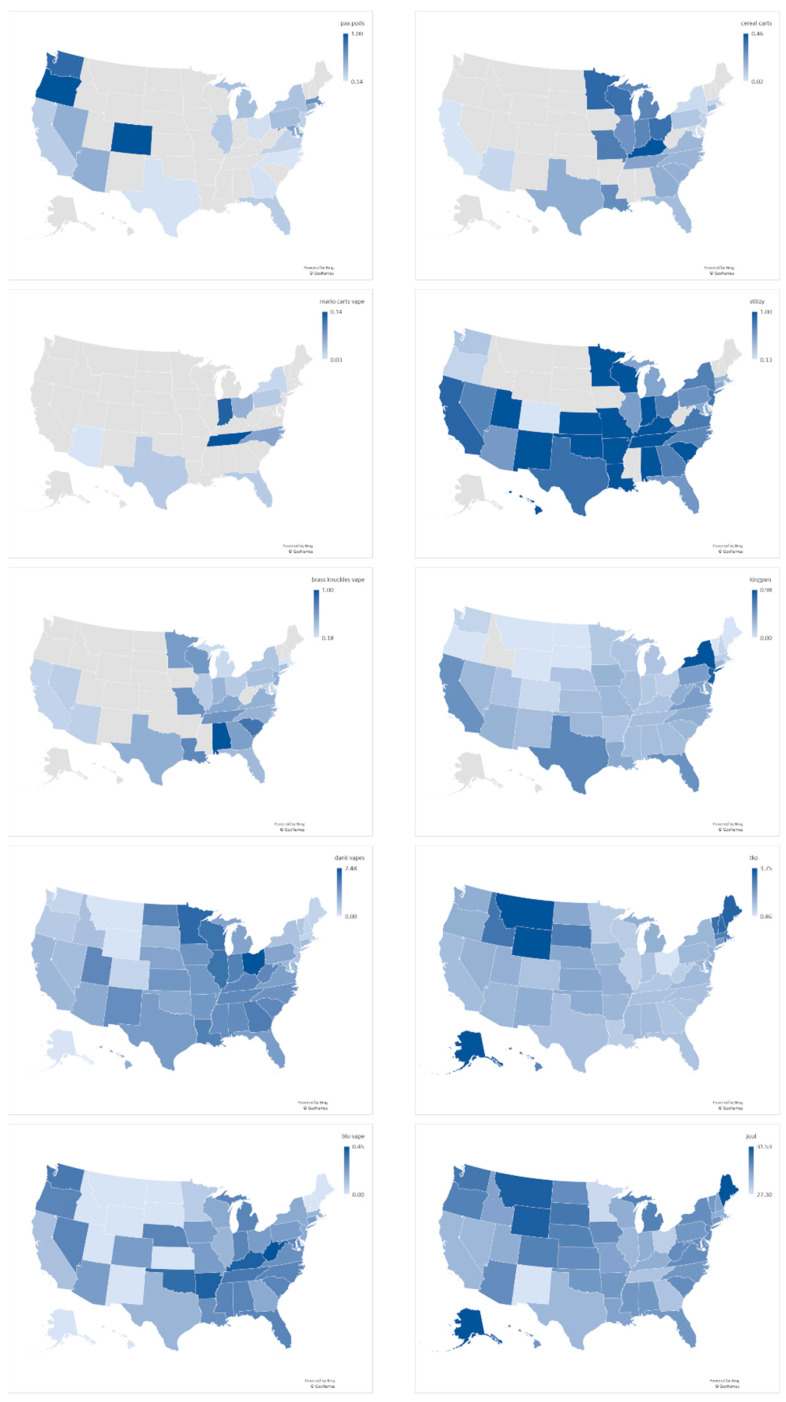
Relative query volume by state, from Google Trends. To facilitate comparison, all query volumes were normalized to the same scale.

Google Trends provides the relative query volume from different states for the different products. Figure 3 shows maps of these query volumes. As the figure demonstrates, while some brands are popular across the US, others are concentrated geographically to specific areas. The correlation between the state-level Google Trends query volume and the fraction of Bing users from each state who queries for each brand were statistically significantly correlated (on average, Spearman rho = 0.66), except for TKO and Blu. We attribute the latter to the fact that these two names are short and ambiguous.

### 3.2. Adverse Reactions to Vaping

YouVape included questions about 12 possible adverse reactions, including: cough, difficulty breathing, chest pain, coughing up blood, nausea, vomiting, diarrhea, stomach pain, fever, chills, feeling tired, and weight loss. These adverse reactions comprised of the reactions reported in the CDC Investigation notice [1], as well as symptoms which were not reported therein and were included in YouVape as control conditions (coughing up blood, stomach pain, and chills) to estimate people’s likelihood of indicating side effects which are unlikely to be caused by vaping.

**Figure 4:**
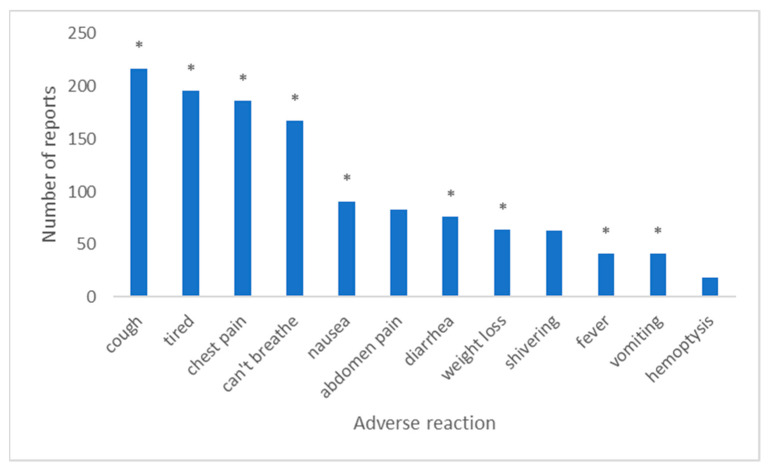
Number of reports for each adverse reaction on YouVape. Stars denote adverse reactions reported by CDC.

Figure 4 shows the number of reports for each adverse reaction from YouVape. As the figure shows, the control reactions received fewer reports than the known reactions, but this difference is not statistically significant (ranksum test, *p* = 0.2).

As noted above, we scored the symptoms on Bing and Reddit. The scores of the symptoms mentioned by at least 1000 Bing users and those of Reddit are correlated (*n* = 55, Spearman rho = 0.29, *p* = 0.03). The correlation between Bing and Reddit scores and the number of reports on YouVape is weak: 0.03 and 0.24 (*n* = 12), respectively.

Previous work has shown that acute reactions might be more likely to be reported in some data sources but not in others (as reported in Yom-Tov and Gabrilovich [32]). Therefore, we followed the methodology reported in [32] (referred therein as Most Discordant Adverse Reactions) and attempted to exclude two adverse reactions which would maximally improve the correlation between YouVape data and (separately) Bing and Reddit. The two reactions for Bing were breathing difficulty and fever (rho = 0.37), and for Reddit difficulty breathing and vomiting (rho = 0.27).

Finally, we modeled the likelihood of reporting each of the adverse reactions on YouVape using a logistic regression model, where the independent attributes were age, gender, vaping duration, vaping frequency and the ingredients (model 1) or products (model 2) reportedly consumed by the participant. Only products and ingredients for which 10 or more reports were available were included in this analysis. The dependent variable was whether a user reported a specific adverse reaction.

The models are shown in Table 2. Statistically significant model parameters in the model indicate an association of several products with specific adverse reactions: Juul with cough, Dank Vape with nausea, and TKO with stomach pain (a control condition). Additionally, CBD is associated with fever, while THC is associated with diarrhea. Finally, younger age is often associated with fewer symptoms. We note that the indicator of whether the respondent had a chronic condition was tested but was not statistically significantly correlated in any of the models. Models for separate products and ingredients are provided in Table A1 and Table A2.

We attempted to identify similar correlations in Reddit and in Bing data through a chi^2^ test. Specifically, a 2 × 2 table was constructed, where the rows correspond to whether the user mentioned the product or not, and the columns to whether they mentioned the adverse reaction or not. The values in each cell correspond to the number of users of that combination. No statistically significant interactions were found in Reddit data. Comparing the control population with users who queried for the product on Bing, the highest ranked symptoms for people who queried for all products except mig21 and cereal carts was cough and (general) pain. For mig21, it was depression and weight loss and for cereal carts cough and depression.

We attempted to identify similar correlations in Reddit data by running a chi^2^ test for whether the user mentioned the product and whether they mentioned the adverse reaction, but no statistically significant interactions were found. We applied the same method for Bing data, comparing the control population with users who queried for the product. The highest ranked symptoms for people who queried for all products except mig21 and cereal carts was cough and (general) pain. For mig21 it was depression and weight loss and for cereal carts cough and depression.

Finally, we tested whether the location of purchase was correlated with greater likelihood of adverse reactions. To do so, we used the data from YouVape and tested for each product the association between reported purchase location and whether the user experienced adverse reactions. The only product where statistically significant association were discovered (chi^2^ test, *p* < 0.05 with Bonferroni correction) was non-brand products (marked by users as “Other”): the percentage of people who reported adverse reactions for those products was 76% when bought from a convenience store or gas station, 64% when bought from friends or family, 58% when purchased at a pharmacy, and 40% when bought online or at unknown locations.

## 4. Discussion

In this study we used multiple data sources to study an outbreak of lung illness and other adverse events associated with the use of electronic cigarettes using multiple online data sources. These data sources differ in their volume, the level of detail they offer, the ability to observe individual users (versus populations), and the types of information they provide (geographical, demographic, etc.). Furthermore, the precision and recall of identified users differs, with, for example, YouVape offering the highest precision but lowest recall among the sources.

Based on our findings, even though there are significant correlations between web platforms, each offers a unique vantage point and assists in filling gaps in information that other platforms are unable to provide. We find large geographic variation across vaping products. Models of participatory surveillance data found specific product and adverse reaction associations. Moreover, cannabidiol was found to be associated with fever, while tetrahydrocannabinol was found to be correlated with diarrhea.

YouVape showed that a majority of users purchase vaping products from sources such as gas stations, family, or friends or from online dealers. This is consistent with CDC national and state data from patient reports and product sample testing linking most EVALI cases with purchases from informal sources such as friends, family or online dealers [34]. On brand and ingredients, all analyzed data sources were broadly in agreement. Paralleling the results of our study, CDC reported that evidence supports that multiple brands were likely responsible for the outbreak [35]. Our results showed that Dank Vapes were widely searched across the USA, congruent with CDC reports documenting these products as the most commonly reported product in major US Census regions [35]. Additionally, Google Trends indications of the popularity of TKO carts in the North West is consistent with CDC reports, which showed TKO more commonly reported by EVALI patients from the western US [35].

Data from YouVape showed that males are more likely to vape, as also found by previous research on vaping [35]. Though YouVape users were typically older than Reddit users, the distribution of ages for both platforms was similar to that reported in national surveys [36].

These parallel findings with CDC reports and past literature, together with the similarities among sources, suggest that digital data streams provide valid information that can be used to uncover real world behavioral trends.

Findings from our study showed that participatory surveillance documented details about vaping not directly captured in other platforms to the same extent, and possibly from a population more strongly affected by the adverse effects of vaping. For example, the frequency of vaping reported on YouVape was much greater than that reported in surveys [36], as was the percentage of users reporting adverse events (60%). This may be because active participation is required from participatory surveillance platforms such as YouVape, which those with vaping-related symptoms are more likely to engage in. Comparing the adverse reactions reported by YouVape users to data from Reddit and Bing, YouVape users reported more acute symptoms that overlapped with symptoms reported in EVALI cases [37,38]. This may be a reflection of the different types of users across platforms or how different methods of retrieval of information, direct versus organic, influence public responses.

Digital surveillance sources can capture information beyond traditional sources of surveillance methods. Although vaping-related symptoms from our study parallel findings from reports from the Center for Disease Control, we also found additional side effects not captured by CDC. These differentially reported symptoms included abdomen pain, shivering, and hemoptysis. It may be the case that these are symptoms related EVALI that CDC did not capture or that these symptoms represent additional (no-EVALI) side effects of vaping. The CDC has yet to draw links between specific brands and EVALI cases. Although Dank Vapes and TKO carts were identified by the CDC as linked with EVALI [34], specific symptoms related to these brands were not documented. In contrast, our data suggests certain brands may be contributing to specific symptoms (e.g., Dank Vape associated with nausea). Additionally, we found certain ingredients to be associated with specific symptoms—i.e., CBD with fever, and THC with diarrhea. This specificity of symptoms, ingredients, and brands warrant further investigation about the additional adverse reactions from vaping of commonly used vaping products. It could also be the case that our population was unrepresented by traditional research because of their access (or lack thereof) to healthcare and illicit nature of these users’ actions [14]. Evidence of this may be shown by our findings that users provided detailed information about their recipes of vaping liquid that they concoct, a fact not often disclosed.

Data retrieved from each of the data sources in this work offer differing, complimentary, information. The two platforms that provided the most detailed description on users and their experiences were YouVape and Reddit. On these platforms we obtained user-level information on demographics (age and gender) as well as vaping-related behaviors such as the type of products and brands users preferred. The anonymity afforded by these platforms may provide users with a high level of comfort at disclosing information [39]. Moreover, online anonymous forums like Reddit provide information about unique emerging trends. However, people in these two sources (participatory surveillance platforms and community forums) may have selection bias, making it unclear as to how pervasive and generalizable observable trends in these populations are to the general population. Additionally, both platforms identify people based on self-identification which is not independently verified. Both platforms (but especially YouVape) are limited in the number of people that utilized them, making the volume of data from these platforms smaller. Finally, participatory surveillance mechanisms can only be set in place for known problems. We could only start YouVape once it was realized that there were vaping-induced health issues.

The ubiquitous nature of search engines such as Bing and Google make their data informative with regards to population-level usage and geographic variation. Additionally, benefits of data we retrieved from Bing is that it allowed linking anonymous individual-level searches. This method enabled us to detect adverse events even when users did not make these associations themselves, in the same way that adverse reactions to pharmaceutical drugs have been detected [32]. Data from Google Trends enabled us to capture widespread patterns of brand usage across the entire United States, which was found consistent with reports derived by the CDC. In contrast with YouVape, these data could be used to earlier detect areas of illicit vaping distribution and use. Therefore, although there are limitations in detail from Google and Bing, advantages of these platforms is volume, reach and generalizability of the findings to populations across the US as well as the ability to mine archival data to detect abnormalities in near real-time [13].

We originally chose three symptoms (coughing of blood, stomach pain, and chills) which did not appear in the CDC report as control symptoms. The rate of reporting on these symptoms on YouVape was not statistically significantly different from that of the known symptoms. Moreover, they were found to be correlated with specific brands and\or components (cannabidiol with coughing of blood, TKO with stomach pain). There could be several reasons for this: First, it may be that people’s reports are noisy. It could also be that these symptoms, although not appearing in the original report, are experienced by people who vape. Therefore, in future, additional symptoms, including both plausible and implausible ones, should be offered as control symptoms. In cases where control symptoms are found to be significantly associated with the substance of interest, further investigation should be conducted to ascertain the reason for the identified association.

Two of our data sources were public (Reddit and Google Trends), one created by the researchers (YouVape) and one was private (Bing). Replication of our results is, however, possible by using similar data sources to that of the private dataset. Such datasets are accessible by researchers, e.g., [40].

Although each data source provides access to potentially different populations and each suffers from respective biases, we note that the correlation of product popularity and of adverse reactions between sources is statistically significant.

## 5. Conclusions

Rapid information collection is required in conjunction with frequent iterations to capture population-level changes that occur during a health outbreak. Digital surveillance sources enable the capturing of unique, organic, and real-time information about such outbreaks, in contrast with more traditional data surveillance, which is limited in its ability to detect novel emerging population trends and is relatively non-adaptive in its collection of information. Participatory surveillance provides a greater level of detail and discrete behaviors, but requires prior knowledge of the need to capture this information as well as people’s knowledge of the platform and willingness to contribute to it. In contrast, passively collected streams such as Google Trends, if monitored, can provide preemptive surveillance at the population level. Combining these resources of active and passive digital data (and of traditional data sources) enables capturing a breadth of information, giving a better picture of the ongoing concern. Maintaining digital cohorts could provide the best of both worlds whereby users provide passive information continuously and active inquiries can be deployed in times of need to obtain in-depth real time information in a rapidly changing environment.

## Figures and Tables

**Table 1:** Summary of data sources.

Source	Number of Users	Date Range	Source Type
YouVape	720	29 October 2019—25 January 2020	Participatory, online digital cohort
Google Trends	Unknown	1 January 2018—31 December 2019	Web search, aggregate
Bing	1.03 M (vaping group), 3.2 M (control group)	1 October 2018—30 June 2019	Web search, anonymous individuals
Reddit	4331	1 January 2015—31 December 2019	Social media, anonymous individuals

**Table 2:** Logistic regression model coefficients for ingredients (top) and products (bottom). Stars denote statistically significant results (*p* < 0.05, with Bonferroni correction for each part of the table separately). Duration refers to the reported duration of vaping.

	Chest Pain	Chills	Cough	Coughing Up Blood	Diarrhea	Difficulty Breathing	Feeling Tired	Fever	Nausea	Stomach Pain	Vomiting	Weight Loss
Age	0.96 *	0.97	0.98	1.01	0.96 *	0.98	0.97 *	0.97	0.97	0.96 *	0.94 *	0.99
Is female	0.83	0.51	0.60	0.23	0.63	0.69	0.80	1.01	0.46	0.63	1.19	0.78
Duration	1.48 *	1.14	1.21	1.97	1.11	1.48 *	1.31	1.20	1.07	1.13	1.28	1.34
Vape freq.	1.21	1.07	1.01	1.04	1.12	1.05	1.28 *	1.11	1.27	1.07	0.91	1.12
CBD	1.39	2.12	1.02	10.70 *	1.35	1.11	1.38	3.82 *	1.65	2.16	2.53	2.39
Flavored	0.79	0.71	0.72	0.31	0.87	0.80	0.98	0.96	0.93	1.21	1.12	0.78
Homemade	0.83	0.73	0.81	5.37	0.80	0.63	0.62	0.79	0.91	1.02	1.46	0.95
Nicotine	1.04	0.68	1.45	5.81	1.02	1.22	0.77	0.80	0.82	1.32	0.63	1.05
Other	2.12	1.99	1.97	1.54	4.39	1.48	1.62	2.25	2.27	3.16	3.86	3.86
THC	1.20	1.57	1.72	1.11	2.46 *	1.62	1.42	1.00	1.88	2.10	2.61	1.82
Model R^2^	0.06	0.03	0.04	0.12	0.03	0.04	0.05	0.02	0.04	0.05	0.05	0.03
Model *p*-value	<10^−4^	0.0002	<10^−4^	0.0001	0.0002	<10^−4^	<10^−4^	0.0536	0.0001	<10^−4^	<10^−4^	0.0027
Age	0.97 *	0.98	0.99	1.01	0.98	0.98	0.98	0.98	0.98	0.97	0.95	1.00
Is female	0.81	0.40 *	0.63	0.20	0.55	0.66	0.74	0.86	0.42 *	0.61	0.87	0.62
Duration	1.43 *	1.14	1.25	1.80	1.07	1.51 *	1.36	1.30	1.13	1.13	1.34	1.31
Vape freq.	1.16	0.97	0.97	0.86	1.05	1.02	1.21	1.01	1.12	0.99	0.79	1.03
blu	1.54	1.42	1.48	10.07	1.65	1.32	1.62	2.05	1.90	1.95	2.64	1.82
Brass knuckles	2.69	2.34	3.00	1.46	2.77	2.48	1.02	0.98	2.23	1.73	1.04	2.51
Cereal carts	1.00	0.93	0.74	4.44	0.38	0.92	0.45	1.20	0.80	2.12	1.05	1.51
Dank vape	2.72	1.31	2.18	4.66	1.82	1.70	2.32	1.86	3.78 *	2.69	2.29	0.95
exotics	2.41	0.23	0.32	0.00	0.68	0.68	0.35	0.53	0.23	0.46	0.73	0.72
Juul	1.45	0.90	2.39	4.06	1.08	1.65	1.04	2.48	1.86	2.39	1.42	1.14
kingpen	0.97	1.32	0.98	0.07	1.28	1.40	1.92	0.70	0.71	0.58	0.92	1.32
Mario carts	0.98	5.75	4.31 *	107.77	3.06	3.03	2.20	6.62	3.13	2.29	3.94	5.87
Other	0.67	0.68	1.05	9.12	0.88	1.02	1.01	1.55	1.73	1.32	1.93	1.26
Pax	0.30	0.76	1.09	2.66	0.79	0.42	0.76	1.67	1.55	1.49	1.70	1.49
Stiiizy	0.55	1.27	0.61	0.86	1.93	1.46	1.46	1.17	0.44	0.68	0.99	1.22
TKO	1.63	2.80	0.93	8.58	3.06	0.90	1.73	1.99	2.51	4.44 *	4.01	0.93
Model R^2^	0.10	0.06	0.07	0.28	0.06	0.06	0.05	0.08	0.07	0.08	0.09	0.03
Model *p*-value	<10^−4^	0.0004	<10^−4^	<10^−4^	0.0001	<10^−4^	<10^−4^	0.0417	<10^−4^	<10^−4^	0.0003	0.0389

## Data Availability

Google Trends and Reddit data are publicly available on their respective websites. Bing and YouVape data are available on reasonable request from the authors, after signing appropriate agreements.

## Appendix A. Logistic Regression Model Coefficients for Separate Ingredients and Product

**Table A1:** Logistic regression model coefficients for separate ingredients. Stars denote statistically significant results (*p* < 0.05, with Bonferroni correction for 6 ingredients and 12 adverse reactions). Duration refers to the reported duration of vaping.

Product	Parameter	Chest Pain	Chills	Cough	Coughing Up Blood	Diarrhea	Difficulty Breathing	Feeling Tired	Fever	Nausea	Stomach Pain	Vomiting	Weight Loss
CBD	Age	−0.04	−0.02	−0.01	−0.01	−0.04	−0.02	−0.04	−0.03	−0.03	−0.03	−0.06	−0.02
	Is female	−0.06	−0.56	−0.23	−0.82	0.26	−0.57	−0.01	−0.24	−1.04	0.10	0.32	0.05
	Duration	0.64	0.50	0.41	0.36	0.11	0.79	0.71	0.27	0.46	0.55	0.13	−0.07
	Vape freq.	0.61	0.41	0.21	0.31	0.19	0.43	0.59	0.25	0.57	0.37	0.06	−0.01
	R^2^	0.00	0.24	0.42	0.60	0.33	0.02	0.00	0.51	0.02	0.08	0.20	0.92
Flavored	Age	−0.06 *	−0.02	−0.05	0.00	−0.07	−0.04	−0.04	−0.04	−0.04	−0.03	−0.04	0.03
	Is female	−0.10	−1.20	−0.66	−1.64	−0.01	−0.99	−0.19	−0.57	−1.52	−0.62	−0.23	−1.04
	Duration	0.48	−0.16	0.10	0.00	−0.44	0.41	0.22	0.13	0.02	0.49	0.14	−0.28
	Vape freq.	0.16	−0.30	−0.06	−0.50	−0.05	0.07	0.14	−0.21	0.05	−0.03	−0.32	−0.36
	R^2^	0.00	0.18	0.00	0.25	0.05	0.00	0.05	0.37	0.01	0.04	0.30	0.12
Homemade	Age	−0.01	0.11	−0.02	0.11	0.01	0.02	0.00	0.11	0.02	0.01	0.05	0.10
	Is female	−2.69	−2.33	−2.54	−2.33	−1.90	−3.10	−2.69	−2.33	−1.64	−1.69	−1.94	−1.54
	Duration	0.78	−74.34	0.5	−74.34	−66.66	0.73	0.55	−74.34	−61.04	−0.14	−67.57	−53.74
	Vape freq.	−0.13	−0.61	0.05	−0.61	0.03	−0.07	0.10	−0.61	0.10	0.26	−0.20	−0.67
	R^2^	0.08	0.1	0.27	0.10	0.5	0.08	0.24	0.10	0.47	0.73	0.35	0.05
Nicotine	Age	−0.05 *	−0.04	−0.04 *	0.02	−0.04	−0.03	−0.03	−0.03	−0.04	−0.04	−0.06	0.00
	Is female	−0.30	−0.65	−0.65	−1.39	−0.70	−0.57	−0.50	−0.59	−1.07 *	−0.64	−0.41	−0.99
	Duration	0.53 *	0.1	0.28	0.69	0.12	0.22	0.18	0.33	0.07	0.21	0.23	−0.18
	Vape freq.	0.10	−0.07	0.02	−0.13	−0.10	−0.12	0.15	0.05	0.17	0.02	−0.11	−0.13
	R^2^	0.00	0.10	0.00	0.01	0.02	0.00	0.00	0.26	0.00	0.02	0.08	0.10
Other products	Age	−0.01	8.89	−0.02	8.89	0.08	0.12	25.4	8.89	0.04	0.09	0.04	16.83
	Is female	−1.16	105.2	−1.02	105.2	1.94	2.08	468.66	105.2	−0.29	1.98	−0.29	−45.57
	Duration	0.32	20.03	0.1	20.03	−58.94	0.79	330.06	20.03	−58.36	−0.13	−58.36	120.54
	Vape freq.	−0.08	−7.17	0.04	−7.17	0.44	0.31	59.45	−7.17	−0.16	0.44	−0.16	−141.9
	R^2^	0.95	0.06	0.99	0.06	0.44	0.61	0.00	0.06	0.52	0.73	0.52	0.01
THC	Age	−0.03	−0.02	−0.02	0.01	−0.04	−0.02	−0.03	−0.01	−0.01	−0.03	−0.05	0.00
	Is female	0.16	−0.58	−0.33	−0.95	0.02	−0.06	−0.09	0.22	−0.48	−0.19	0.56	−0.11
	Duration	0.26	0.3	0.12	0.32	0.13	0.52	0.36	0.18	0.11	0.05	0.21	0.30
	Vape freq.	0.22	0.05	0.11	0.12	0.18	0.04	0.27	0.01	0.22	0.17	−0.23	0.08
	R^2^	0.00	0.15	0.20	0.49	0.04	0.00	0.00	0.94	0.27	0.13	0.02	0.67

**Table A2:** Logistic regression model coefficients for separate products. Stars denote statistically significant results (*p* < 0.05, with Bonferroni correction for 12 brands and 12 adverse reactions). Duration refers to the reported duration of vaping.

Product	Parameter	Chest Pain	Chills	Cough	Coughing Up Blood	Diarrhea	Difficulty Breathing	Feeling Tired	Fever	Nausea	Stomach Pain	Vomiting	Weight Loss
Blu	Age	0.01	0.06	−0.02	0.02	0.00	0.00	0.01	0.02	0.00	−0.04	0.03	−0.04
	Is female	0.44	−3.40	0.11	−1.79	−0.96	−0.54	−1.35	−1.55	−2.18	−1.39	−0.57	−56.66
	Duration	0.62	1.17	0.09	0.45	−0.21	0.74	0.67	0.50	0.36	−0.15	0.11	−108.33
	Vape freq.	0.20	−0.45	−0.50	−0.32	−0.09	−0.49	−0.23	−0.09	0.10	0.07	−0.17	−54.95
	R^2^	0.79	0.03	0.56	0.43	0.90	0.23	0.30	0.49	0.16	0.56	0.92	0.00
Brass	Age	0.05	0.05	0.04	12.08	0.05	0.07	0.06	0.12	0.06	0.04	0.06	0.06
knuckles	Is female	−1.61	−1.03	−0.26	120.68	1.21	−0.16	0.64	0.99	0.60	0.63	0.97	0.89
	Duration	0.63	0.11	−0.32	80.05	−0.01	0.54	0.84	0.68	0.26	−0.03	0.23	−0.39
	Vape freq.	0.10	0.07	−0.19	0.14	0.15	−0.02	0.22	0.11	0.40	0.18	0.25	−0.36
	R^2^	0.25	0.63	0.75	0.03	0.39	0.39	0.22	0.32	0.44	0.76	0.61	0.46
Cereal carts	Age	15.67	24.72	0.34	24.72	24.72	0.33	0.37	24.72	24.72	5.15	24.72	3.61
	Is female	454.87	308.89	10.14	308.89	308.89	8.37	9.61	308.89	308.89	−118.46	308.89	104.09
	Duration	−24.78	73.78	−1.24	73.78	73.78	−0.59	−0.80	73.78	73.78	87.39	73.78	−349.42
	Vape freq.	102.92	83.1	2.41	83.1	83.1	0.15	2.33	83.1	83.1	−118.78	83.10	−198.27
	R^2^	0.02	0.01	0.17	0.01	0.01	0.12	0.18	0.01	0.01	0.00	0.01	0.03
Dank vapes	Age	0.03	0.07	0.05	0.11	0.00	0.03	0.03	0.08	0.03	0.03	0.01	0.04
	Is female	1.63	0.18	0.27	1.34	1.16	0.27	−0.52	0.06	0.40	1.41	1.96	2.40
	Duration	−0.26	0.33	−0.50	−69.38	−0.17	0.11	0.61	0.60	−0.21	−0.07	−1.1	−0.18
	Vape freq.	0.76	0.34	−0.02	−0.35	0.02	0.29	0.49	0.10	0.03	−0.13	−0.54	−0.02
	R^2^	0.05	0.12	0.22	0.02	0.63	0.62	0.17	0.10	0.83	0.43	0.11	0.29
Exotics	Age	0.03	0.10	0.03	8.70	0.05	0.05	0.13	0.08	0.08	0.01	0.00	0.07
	Is female	2.73	0.09	1.23	64.73	0.75	1.99	3.28	0.63	0.72	−1.53	−2.03	1.06
	Duration	−0.56	−0.22	−0.79	−43.39	−0.82	−0.34	−0.3	−0.16	−0.47	−0.39	−0.27	−0.65
	Vape freq.	0.43	−0.03	0.35	19.58	0.10	0.59	0.91	0.23	0.22	−0.2	−0.41	0.37
	R^2^	0.23	0.41	0.39	0.01	0.49	0.51	0.26	0.70	0.59	0.65	0.50	0.56
Juul	Age	−0.04	−0.02	0.00	0.05	−0.01	−0.05	−0.03	−0.01	−0.06	−0.04	−0.01	0.03
	Is female	0.14	0.01	−0.29	−1.25	−0.82	−0.07	−0.76	−0.17	−0.6	−0.47	−0.27	−1.07
	Duration	0.72	0.19	0.27	0.43	0.37	0.05	0.46	0.24	0.03	0.16	−0.12	−0.44
	Vape freq.	0.37	−0.01	0.18	−0.28	0.05	−0.18	0.46	0.05	0.15	0.10	−0.37	−0.11
	R^2^	0.01	0.90	0.56	0.14	0.23	0.03	0.01	0.96	0.06	0.32	0.51	0.12
Kingpen	Age	−0.01	0.08	0.03	0.3	0.03	0.04	0.04	0.17	0.04	0.02	0.05	0.04
	Is female	0.51	0.71	−0.49	6.28	1.14	0.43	1.32	2.56	0.19	0.04	1.13	0.06
	Duration	0.21	0.43	−0.03	0.98	0.01	0.38	0.85	0.82	0.14	0.03	0.29	0.29
	Vape freq.	0.05	−0.03	0.35	0.11	−0.06	0.04	0.20	0.32	0.39	−0.16	−0.16	0.10
	R^2^	0.84	0.18	0.53	0.01	0.67	0.57	0.06	0.01	0.58	0.97	0.64	0.70
Mario carts	Age	0.07	0.12	0.12	0.22	0.17	0.21	0.1	0.12	0.12	0.22	0.12	0.09
	Is female	2.70	2.34	7.09	4.27	4.52	6.78	3.47	2.34	2.34	4.21	2.34	1.80
	Duration	0.91	0.96	−0.43	0.4	0.98	−0.27	0.33	0.96	0.96	2.23	0.96	−0.08
	Vape freq.	0.84	0.88	1.84	1.22	1.49	0.79	0.98	0.88	0.88	1.01	0.88	−0.06
	R^2^	0.63	0.40	0.15	0.20	0.23	0.16	0.49	0.40	0.40	0.09	0.40	0.74
Other	Age	−0.03	−0.01	−0.03	0.02	−0.03	−0.01	−0.03	−0.01	−0.02	−0.03	−0.03	0.00
	Is female	−0.09	−1.32	−0.82	−2.12	−0.73	−0.63	−0.23	−0.70	−1.34 *	−0.83	−0.20	−0.41
	Duration	0.37	0.11	0.27	0.43	−0.12	0.51	0.39	0.10	0.01	−0.12	0.36	0.45
	Vape freq.	0.04	−0.1	−0.02	−0.06	0.04	0.07	0.02	−0.09	0.15	0.06	−0.13	0.15
	R^2^	0.01	0.02	0.00	0.00	0.10	0.00	0.00	0.54	0.00	0.08	0.14	0.21
Pax	Age	−0.01	0.02	−0.02	0.04	0.01	0.01	−0.03	0.02	−0.03	−0.02	0.02	0.03
	Is female	−0.87	−0.59	−2.00	−1.01	0.06	−1.84	−1.64	−0.59	−1.77	−0.89	−0.41	1.69
	Duration	0.61	0.95	0.31	0.87	0.52	1.22	0.54	0.95	0.73	0.59	0.71	0.29
	Vape freq.	0.43	0.40	0.46	0.46	0.40	0.96	0.81	0.40	0.68	0.61	0.34	−0.24
	R^2^	0.59	0.41	0.06	0.48	0.73	0.06	0.05	0.41	0.08	0.29	0.67	0.39
Stiiizy	Age	0.07	0.10	0.16	6.8	0.03	0.06	0.06	0.13	0.13	0.11	0.13	0.24
	Is female	1.05	−0.06	−5.39	−67.77	1.68	−0.35	−0.04	0.62	0.62	−2.08	0.62	−5.56
	Duration	−0.05	−0.26	−62.64	−2.12	−1.27	0.45	−0.22	0.17	0.17	−63.68	0.17	1.23
	Vape freq.	0.31	−1.07	0.58	−39.8	0.15	−0.51	0.94	−0.81	−0.81	0.33	−0.81	−0.38
	R^2^	0.38	0.20	0.01	0.01	0.37	0.51	0.19	0.17	0.17	0.12	0.17	0.08
TKO	Age	−0.01	0.00	0.00	0.07	0.01	0.03	0.00	0.06	0.03	−0.01	0.01	0.05
	Is female	0.34	−1.29	0.46	−0.53	−0.24	0.25	0.30	−0.24	−0.03	−0.13	−0.33	−0.78
	Duration	0.37	0.40	0.34	0.12	0.15	0.52	0.80	0.40	0.65	0.03	0.26	0.02
	Vape freq.	0.7	0.01	0.10	0.03	0.14	0.63	0.77	−0.13	0.34	0.00	−0.27	−0.02
	R^2^	0.25	0.43	0.87	0.45	0.98	0.37	0.14	0.55	0.43	1.00	0.81	0.46
